# Acceptance of Rubella-Containing Vaccine and Factors Associated with Acceptance among Women of Reproductive Age in China: A Cross-Sectional Study

**DOI:** 10.3390/vaccines12101153

**Published:** 2024-10-08

**Authors:** Xia Xu, Siyu Liu, Xiaoqi Wang, Aodi Huang, Jun Li, Yang Zhou, Lei Wang, Yong Qin, Yu Ma, Shuyi An, Jun Liu, Lin Tang, Zundong Yin, Fuzhen Wang

**Affiliations:** 1 National Immunization Program, Chinese Center for Disease Control and Prevention, Beijing 100050, China; xuxia@chinacdc.cn (X.X.); liusy@chinacdc.cn (S.L.); wangxq1@chinacdc.cn (X.W.); huangad@chinacdc.cn (A.H.); tanglin@chinacdc.cn (L.T.); yinzd@chinacdc.cn (Z.Y.); 2 National Key Laboratory of Intelligent Tracking and Forecasting for Infectious Diseases, Chinese Center for Disease Control and Prevention, Beijing 102206, China; 3 Henan Provincial Center for Disease Control and Prevention, Zhengzhou 450016, China; henan_epi2@163.com; 4 Zhejiang Provincial Center for Disease Control and Prevention, Hangzhou 310051, China; yzhou@cdc.zj.cn; 5 Hubei Provincial Center for Disease Control and Prevention, Wuhan 430079, China; 18627940191@163.com; 6 Sichuan Provincial Center for Disease Control and Prevention, Chengdu 610041, China; 18081882321@163.com; 7 Shaanxi Provincial Center for Disease Control and Prevention, Xian 710054, China; mayu.u@163.com; 8 Liaoning Provincial Center for Disease Control and Prevention, Shenyang 110005, China; anshuyi1981@163.com; 9 Guangdong Provincial Center for Disease Control and Prevention, Guangzhou 511430, China; varrt@163.com

**Keywords:** women of reproductive age, rubella-containing vaccine, acceptance, structural equation model

## Abstract

**Objectives**: To investigate the acceptance and factors influencing acceptance of rubella-containing vaccine (RCV) among women of reproductive age in Guangdong, Henan, Hubei, Liaoning, Shanxi, Sichuan and Zhejiang provinces of China. **Methods**: Using a stratified sampling method, we selected one urban and one rural community health services center in each of two cities in seven provinces. From these centers, we recruited women 15–49 years of age to complete a questionnaire on their willingness to receive RCV and factors influencing willingness. The survey instrument was adapted from the behavioral and social drivers (BeSD) of vaccination survey tool developed by the World Health Organization. **Results**: Among 1286 participants, 981 (76.3%) were willing to receive RCV. Awareness of rubella ranged from 12.4% to 70.6%. Willingness to receive RCV differed significantly by region, occupation, vaccination history, and awareness. All latent variables of the structural equation model (SEM) were positively correlated with willingness, and all standardized paths were statistically significant (*p* < 0.001). Thinking and Feeling had direct positive effects on Social Processes (*β* = 0.789) and Practical Issues (*β* = 0.542), thereby indirectly affecting motivation. **Conclusions**: Women of reproductive age had high willingness to receive the rubella vaccination, but their general awareness of rubella was relatively low. It is necessary to strengthen the health education of women of reproductive age regarding rubella to improve RCV coverage.

## 1. Introduction

Rubella is a highly contagious respiratory-spread infectious disease most commonly seen in unvaccinated children and adolescents [1]. Rubella virus infection during early pregnancy can cause miscarriage, stillbirth, and a constellation of congenital disorders known as congenital rubella syndrome (CRS), characterized by congenital heart disease, hearing impairment, cataracts, and diminished fetal brain development [2]. Since RCV was introduced nationwide into China’s Expanded Immunization Program (EPI) in 2008, the incidence of rubella has decreased significantly year by year [3], maintaining low levels in recent years (less than 1.0 per million population since 2019). However, rubella IgG seroprevalence among women of reproductive age in China remains relatively low. Population-based serological surveys [4,5,6,7] have consistently shown seroprevalences below 85%, often considered a threshold for herd immunity. Most women of reproductive age in China were born before the 2008 nationwide RCV introduction, and few received RCV through the private market before national introduction. Low seroprevalence in this age group poses a contemporary risk of CRS from indigenous rubella virus and a CRS risk from importation outbreaks, even after indigenous rubella virus is eliminated.

To provide direct protection against rubella, the World Health Organization (WHO) recommends that non-pregnant unvaccinated or seronegative women of reproductive age receive one dose of RCV [8]. The RCV vaccination rate among women of reproductive age in China is low, and previous research [9,10] has shown a low acceptance of RCV in this group. It is therefore essential to explore factors influencing willingness to receive RCV in this key population.

In 2022, the WHO published guidance and tools to investigate behavioral and social drivers (BeSD) influencing vaccination decision-making [11]. Based on the Theory of Planned Behavior, the Health Belief Model, the “3Cs” model of vaccine hesitancy, and other theoretical frameworks, the BeSD framework has four measurable domains addressing vaccine uptake: Thinking and Feeling, Social Processes, Motivation, and Practical Issues. BeSD has been used by many countries to investigate factors related to childhood immunization [12], influenza vaccination [13], and COVID-19 vaccination [14]. We report an investigation into attitudes and influencing factors for RCV vaccination among women of reproductive age in China using an adaptation of the BeSD framework, with the aim of providing evidence for developing strategies and targeted interventions to increase RCV uptake in this population.

## 2. Materials and Methods

### 2.1. Setting Design and Respondents

This was a multi-center cross-sectional study conducted among women of reproductive age on the Chinese mainland. We used a multi-stage stratified sampling method to select two provinces in the eastern region (Zhejiang and Guangdong), the central region (Henan and Hubei) and the western region (Sichuan and Shanxi), and one province in the northeastern region (Liaoning), which can represent these respective regions in economic development and health service availability. From each province, we selected two cities (a provincial capital city and one other city), and in each city, we randomly selected one urban and one rural community health services center or hospital as a study site. Recruitment notices posted at study sites and online publicity were used to inform potential subjects of the specific content and details of the questionnaire and the times designated for the survey. With assistance from provincial centers for disease control and prevention (CDC) staff and physicians, we recruited women of 15–49 years of age from these centers between November 2023 and June 2024 for a face-to-face survey to determine RCV acceptance and factors associated with acceptance. Individuals with hearing or intellectual impairments, or those with severe physical or mental illnesses, were excluded. Participants were recruited into three age-group strata to ensure a balanced distribution across age categories.

Target sample sizes were based on a previous study indicating a 76% acceptance (*p*) of RCV by couples in a preconception care program [15]. We estimated the effect size, with an alpha of 0.05 and a power of 95%. Permissible error (d) was calculated as 0.1 × *p*. The minimum sample size was determined to be 121 respondents per province. To account for attrition, we increased the sample size by 20%, resulting in a target of 145 respondents per province—a total of 1015 respondents.

The study was approved by the Ethical Review Committee of the Chinese Center for Disease Control and Prevention (China CDC) (no. 202401), and was conducted according to the Helsinki Declaration. Written and signed informed consent was obtained from all participants prior to study procedures; for individuals under the age of 18 years, consent was obtained from their guardians.

### 2.2. Survey Instrument

The questionnaire was adapted from WHO’s BeSD tools and guidelines [11] and was divided into four sections: (1) socio-demographic characteristics, including name, date of birth, household registration, occupation, education level, marital status, and childbearing status; (2) rubella infection and RCV vaccination history, (3) knowledge of rubella and RCV vaccination, and (4) the four BeSD drivers.

Knowledge of rubella and RCV was assessed by nine questions with possible responses of yes, not sure, and no. Correct answers scored one point; incorrect answers and not-sure scored zero points. Summed scores were categorized by tertile: low, moderate, and high. The BeSD framework has four dimensions assessed with sixteen questions—four on Thinking and Feeling, four on Social Processes, two on Motivation, and six on Practical Issues. Thinking and Feeling, Social Processes, and Practical Issues were measured with a five-point Likert scale: totally agree, agree, not sure, disagree, and totally disagree. The primary outcome—motivation to receive RCV—was assessed with two yes/no/not-sure questions: intention to get vaccinated, and willingness to recommend RCV to others.

The Cronbach’s α value of all variables was 0.875. The Kaiser–Meyer–Olkin (KMO) value of the questionnaire was 0.897 (Bartlett sphericity test χ^2^ = 9436.37, *p* < 0.001), indicating good reliability and validity. The awareness rate was defined as the ratio of the number of people who were aware of rubella or RCV to the total number of people surveyed. The RCV vaccination willingness rate was the ratio of the number of people willing to receive the RCV to the total number of people surveyed.

### 2.3. Statistical Analyses

Data were collated with EpiData 3.1 and descriptive analyses were performed with SAS 9.4. Quantitative data were expressed as mean ± standard deviation, and categorical variables were described using frequencies and percentages. BeSD responses were combined into two categorical variables (agree and disagree); the Chi-squared test was used for comparisons. Knowledge scores exhibited a skewed distribution and are described using median (M) and interquartile range (IQR). A rank sum test was used for comparisons between groups. Two-sided *p*-values less than 0.05 indicated statistical significance.

All statistically significant variables in the Chi-squared test were included in the structural equation model (SEM). Knowledge of rubella is incorporated as a part of Thinking and Feeling. Confirmatory factor analysis (CFA) was performed to validate the BeSD model of the questionnaire. Items with factor loading <0.4 were excluded from the CFA. An SEM was established with Thinking and Feeling, Social Processes, Motivation, and Practical Issues as four latent variables, with respective observed indicators as observed variables. The overall model fit was evaluated by maximum likelihood (ML) with six indicators. Goodness-of-fit index (GFI), adjusted goodness-of-fit index (AGFI), incremental fit index (IFI), Tucker–Lewis index (TLI) and comparative fit index (CFI) >0.9, and root mean square error of approximation (RMSEA) <0.08, were used to indicate acceptable criteria [16].

## 3. Results

### 3.1. Characteristics of Respondents

As shown in Table 1, 1286 eligible respondents were recruited in this study. Their average age was 33.72 ± 9.81 years; 27.4% were medical practitioners; 52.5% lived in an urban area; and 58.6% had a bachelor’s degree or above. Most were married (70.0%) and had children (66.6%). Monthly incomes of less than CNY 3000, CNY 3001–5000, CNY 5001–10,000, and over CNY 10,000 accounted for 15.9%, 35.5%, 29.9%, and 18.7% of participants, respectively. In total, 123 (14.3%) had a history of adverse pregnancy, including miscarriage, stillbirth, low-birth-weight infants, and other outcomes. Few (3.0%) had a history of rubella infection, and 12.9% had received ≥1 RCV doses; 100 (11.7%) had been tested for rubella IgM, among which 13 (13.0%) were positive.

Among all respondents, 981 (76.3%) were willing to receive RCV, and 940 (73.1%) were willing to recommend RCV to others. Chi-squared testing showed that region, occupation, vaccination history, and rubella knowledge were associated with respondents’ vaccination choice (Table 1).

### 3.2. Awareness and Knowledge

Table 2 shows awareness and knowledge. Rubella awareness for each question ranged from 12.4% to 70.6%, with a median (P_25_–P_75_) rubella knowledge score of 5 (2–7) points over 5 points. Respondents had a relatively high level of awareness regarding contagiousness, clinical manifestations, severe consequences of rubella, and the effectiveness of the measles–mumps–rubella (MMR) vaccine (>50%); 12.4% of respondents were aware that rubella during pregnancy does not necessarily require termination. In addition to a history of adverse pregnancy outcome, there were significant differences in rubella knowledge scores among respondents with different characteristics, including age group, region, household registration, occupation, education level, monthly income, and marital status (Table S1).

### 3.3. Factors Associated with BeSD

**Figure 1:**
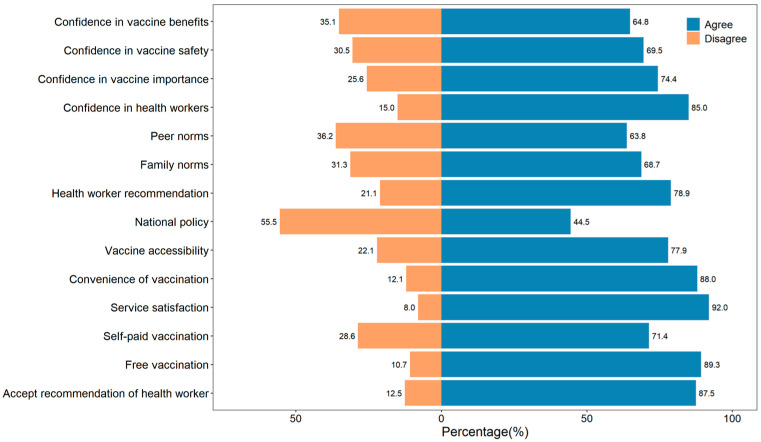
Thinking and Feeling, Social Processes and Practical Issues of respondents’ vaccination acceptance.

As shown in Figure 1 and Table S2, each item of Thinking and Feeling, Social Processes, and Practical Issues had statistically significant association with vaccination acceptance (*p* < 0.001). In the dimension of Thinking and Feeling, 64.9% of respondents believed the vaccine to be effective, 69.5% considered it safe, 74.4% deemed vaccination important, and 85.0% trusted vaccine-related advice from healthcare providers. In Social Processes, vaccination acceptance rates were influenced positively as follows: 63.8% by peers, 68.7% by family, 79.0% by health workers, and 44.5% by national policy. For Practical Issues, vaccine acceptance rates were 77.9% when the vaccine is accessible, 87.9% when vaccination is convenient, 92.0% when services are satisfactory, and 87.5% when recommended by doctors. Additionally, the vaccination acceptance rate increased from 71.4% for self-funded vaccination to 89.3% for government-funded vaccination (Figure 1).

### 3.4. Model Testing with SEM

An SEM was used to construct relationships among four latent variables. Table S3 shows details of the latent variables, their corresponding observed variables, and the values assigned to each observed variable. Based on factor loading, the item “national policy” was ultimately deleted (Table S4). The final model fit indices were GFI = 0.930, AGFI = 0.903, IFI = 0.927, TLI = 0.909, CFI = 0.926, and RMSEA = 0.070, indicating a good overall fit. Tables S4 and S5 show that the questionnaire had good convergence validity and discriminative validity.

**Figure 2:**
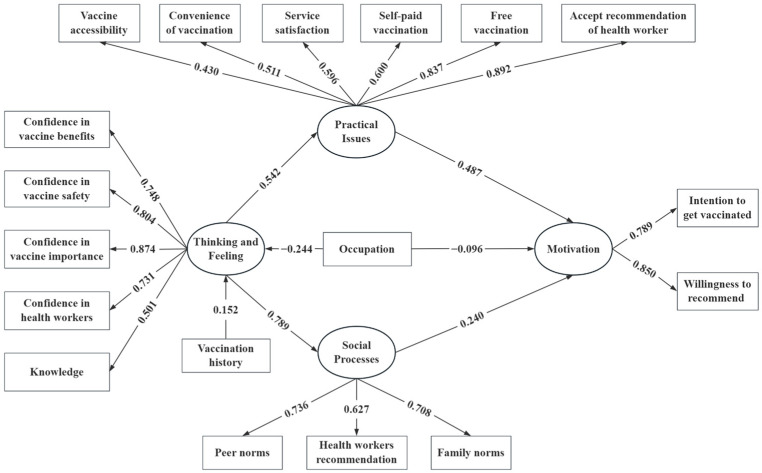
The final integrated SEM with sociodemographic and BeSD effects on the acceptance of RCV.

Relationships between latent variables and corresponding observed variables in the measurement model were all statistically significant. Figure 2 shows the final integrated SEM, while Table 3 explains the specific parameters of each path in the SEM. Social Processes (*β* = 0.240, *p* < 0.001) and Practical Issues (*β* = 0.487, *p* < 0.001) were significantly associated with vaccination motivation. Thinking and Feeling had direct positive effects on Social Processes (*β* = 0.789, *p* < 0.001) and Practical Issues (*β* = 0.542, *p* < 0.001), thereby indirectly affecting motivation. Occupation (*β* = −0.244, *p* < 0.001) and vaccination history (*β* = 0.152, *p* < 0.001) had an effect on Thinking and Feeling. Additionally, compared to medical practitioners, non-healthcare workers expressed a lower acceptance of RCV (*β* = −0.096, *p* < 0.001).

## 4. Discussion

Our cross-sectional survey in seven provinces of China investigating the RCV uptake intentions of women of reproductive age found high RCV acceptance but relatively low rubella-related knowledge. Region, occupation, vaccination history, rubella knowledge, and behavior and social drivers of vaccination factors Thinking and Feeling, Social Processes, and Practical Issues were all associated with RCV acceptance.

Many countries have implemented RCV strategies that include routine childhood immunization combined with the vaccination of rubella-susceptible women of reproductive age. For example, Italy introduced a vaccination strategy targeting young girls and immigrant women [17]. In the United Kingdom, RCV was recommended for adolescents 11–14 years of age and subsequently expanded to include high-risk groups such as nurses and doctors after 1972 [18]. In China, some provinces have adopted a three-dose RCV vaccination strategy for certain populations. For example, Beijing introduced an additional RCV dose for 6-year-old children in 2006, resulting in a significant increase in rubella IgG antibody positivity [19]. Since 2008, Zhejiang province has provided a third dose of MMR vaccine to secondary school students aged 15–19 years, leading to a decline in the incidence and overall proportion of rubella cases in this vaccine target age group [4].

Our finding that three-fourths (76%) of women of reproductive age indicated acceptance of RCV, which is a much higher acceptance rate compared with previous surveys, such as 9% of preconception women in Yiwu [10] and 12% of women of reproductive age in four provinces [9]. In contrast, acceptance rates are notably higher among couples in preconception care programs, reaching 83–89% [15,20]. However, RCV uptake intention is still relatively low. Compared to the general population, women of reproductive age, and particularly pregnant women, have lower acceptance for vaccines against illnesses such as seasonal influenza and COVID-19 [21,22]. Region, occupation, and rubella knowledge were associated with the acceptance of RCV among women of reproductive age, consistent with the existing literature [23].

Rubella knowledge was a key determinant of RCV vaccination among women of reproductive age. However, our finding that rubella knowledge is insufficient, especially regarding RCV vaccination, shows that education is necessary. Other studies [24,25] found low levels of rubella awareness among women of reproductive age and in preconception care programs, implying a lack of adequate knowledge of the potential risks of rubella and CRS. Rubella health education should be conducted to enhance awareness of rubella prevention.

Thinking and Feeling can influence vaccination motivation through Social Processes. Doubt about the effectiveness of the vaccine and inattention to the necessity of vaccination can decrease willingness. Health workers, as a trusted source of information, immensely influence RCV vaccination behavior among women of reproductive age through their knowledge, attitudes, and practices [24]. Thus, integrating rubella vaccination into preconception screening programs and emphasizing the educational role of health workers could enhance the effectiveness of rubella-related health initiatives and boost RCV vaccination rates. Positive social processes can effectively break the “information cocoon” and prevent individuals from remaining vaccine hesitant due to personal cognition and emotions [13]. Respondents were influenced by trusted individuals, including parents, friends, and peers [26,27,28]. A study in the United States showed a significant statistical association between students’ vaccination willingness during the H1N1 influenza pandemic and the attitudes of people in their social networks [29]. Although the influence of national policies was not explored in our SEM, 31% of respondents agreed or somewhat agreed with the statement “The government does not mandate RCV vaccination for women of reproductive age, so it is unnecessary to get vaccinated.” This reflects how governmental endorsement can significantly influence individual decisions [30]. The absence of clear policy directives may diminish the awareness of the importance of rubella prevention in people and reduce the motivation to seek vaccination.

Thinking and Feeling positively affected Practical Issues, which in turn influenced Motivation. Respondents’ answers were influenced by their past vaccination experiences. A satisfying past vaccination experience enhances the willingness to accept a vaccine, whereas experiences such as unsuccessful appointments for HPV vaccine have led to perceptions of insufficient vaccine availability. Several studies have demonstrated that free vaccination policies play a pivotal role in increasing vaccination rates, and regions implementing free vaccination policies achieving higher vaccination rates compared to those without such policies, which is consistent with our results [31,32].

Pregnancy is a contraindication to live vaccines, including all RCVs. Direct protection from CRS can only come from vaccination in advance of pregnancy, but indirect protection against rubella and CRS comes from a high population immunity against rubella virus. Our finding of the positive association between knowledge and acceptance indicates that active promotion and education about rubella and RCV may increase willingness to be vaccinated. Existing studies [33,34] demonstrated essentially no risk of CRS associated with administering rubella vaccine shortly before or during pregnancy. To reduce the immunity gap among women of reproductive age and lower the risk of CRS outbreaks, unvaccinated or rubella-susceptible adolescents and young women can be safely and effectively vaccinated only after verbal screening for pregnancy or plans for pregnancy in the next three months.

Our study has several advantages. To account for variations in economic conditions and healthcare coverage, we used a multistage stratified sampling method to select participants from diverse regions and household registrations across China. We also employed the BeSD survey tools developed by the WHO to investigate social factors and practical issues affecting vaccination. Given the serious consequences of rubella during pregnancy, understanding vaccine uptake in this population is essential for preventing such complications. Additionally, this research provides valuable data to support supplementary immunization activities and guide public health initiatives.

Our study has limitations. Since rubella vaccine was included in the immunization program in China in 2008, RCV vaccination history for older age groups was not available to us. Therefore, the rubella infection history and vaccination status in this study relied on respondents’ recall, which may have caused potential bias. Secondly, the respondents were selected through convenience sampling at the study sites in seven provinces, which may impact the representation of the population. Therefore, caution should be exercised when generalizing our results beyond the specific contexts of this study.

## 5. Conclusions

In conclusion, we found that 76% of women of reproductive age are willing to receive RCV, but overall rubella and RCV knowledge is relatively low, suggesting that there is room for improvement in rubella education in women of reproductive age. The BeSD survey tool showed that willingness to be vaccinated was positively associated with Thinking and Feeling, Social Processes, and Practical Issues. Targeted measures are needed to address these key factors to increase RCV acceptance and vaccination among non-pregnant women of reproductive age.

## Figures and Tables

**Table 1:** Characteristics of respondents (n = 1286).

Characteristic	Total		Willing to Be Vaccinated		χ2	*p*-Value
	N	%	N	%		
Region					43.80	<0.001
Eastern region	365	28.4	273	74.8		
Central region	352	27.4	230	65.3		
Western region	366	28.4	313	85.5		
Northeast region	203	15.8	165	81.3		
Household registration					0.16	0.685
Urban area	675	52.5	518	76.7		
Rural area	611	47.5	463	75.8		
Age					5.67	0.059
15–24 years	264	20.5	214	81.1		
25–34 years	464	36.1	340	73.3		
35–49 years	558	43.4	427	76.5		
Occupation					15.41	<0.001
Medical practitioner	353	27.4	296	83.9		
Not medical practitioner	933	72.6	685	73.4		
Education level					0.37	0.541
High school and below	533	41.4	402	75.4		
Bachelor’s degree and above	753	58.6	579	76.9		
Monthly income in CNY					5.19	0.159
≤3000	205	15.9	147	71.7		
3001–5000	456	35.5	356	78.1		
5001–10,000	384	29.9	301	78.4		
>10,000	241	18.7	177	73.4		
Marital status					0.87	0.349
Married	900	70.0	680	75.6		
Unmarried	386	30.0	301	78.0		
Childbearing status					0.64	0.424
Yes	857	66.6	648	75.6		
No	429	33.4	333	77.6		
Number of children					2.74	0.098
One	473	55.2	368	77.8		
Two and above	384	44.8	280	72.9		
Adverse pregnancy history					0.83	0.364
Yes	123	14.3	89	72.4		
No	734	85.7	559	76.2		
Rubella infection					2.41	0.120
Yes	38	3.0	33	86.8		
No or not sure	1248	97.0	948	76.0		
Rubella vaccine					9.03	0.003
Vaccinated	166	12.9	142	85.5		
Unvaccinated or not sure	1120	87.1	839	74.9		
Knowledge score					89.40	<0.001
Low	394	30.6	238	60.4		
Middle	508	39.5	403	79.3		
High	384	29.9	340	88.5		

**Table 2:** Number of correct responses and awareness rate for rubella knowledge.

Item	Number of Correct Responses	Awareness Rate (%)
Rubella is a highly contagious viral infection.	908	70.6
Infected with rubella can cause fever, rash and other symptoms.	903	70.2
Rubella infection in the first trimester may result in congenital heart defects, blindness, cataracts, hearing impairments, and other birth defects.	741	57.6
Receiving MMR vaccine can effectively prevent mumps, measles, and rubella.	790	61.4
MMR vaccine is not only administered to children.	498	38.7
Prior infection with rubella or vaccination with MMR vaccine greatly reduces the likelihood of contracting the disease again.	489	38.0
It is recommended to not attempt pregnancy for the 3 months following RCV vaccination.	549	42.7
Rubella infection during pregnancy does not require termination of pregnancy.	160	12.4
Women of reproductive age (15–49 years old) can receive the rubella vaccine at vaccination clinics.	495	38.5

**Table 3:** Path diagram of Thinking and Feeling, Social Processes, Motivation, and Practical Issues.

Path	Standard Regression Coefficient (*β*)	S.E. ^1^	C.R. ^1^	*p*-Value
Occupation → Thinking and Feeling	−0.244	0.008	−7.821	<0.001
Vaccination history → Thinking and Feeling	0.152	0.066	4.976	<0.001
Thinking and Feeling → Social Processes	0.789	0.038	20.434	<0.001
Thinking and Feeling → Practical Issues	0.542	0.025	11.679	<0.001
Practical Issues → Motivation	0.487	0.043	9.730	<0.001
Social Processes → Motivation	0.240	0.018	6.270	<0.001
Occupation → Motivation	−0.096	0.003	−3.646	<0.001

^1^ C.R., critical ratio; S.E., standard error.

## Data Availability

Data will be available on reasonable request by contacting the corresponding author.

## Supplementary Materials

The following supporting information can be downloaded at: https://www.mdpi.com/article/10.3390/vaccines12101153/s1, Table S1: Rubella knowledge score of respondents; Table S2: Comparison of RCV acceptance in the dimension of Thinking and Feeling, Social Processes, and Practical Issues; Table S3: Associations and values between latent variables and corresponding observed variables; Table S4: Factor loading and convergent validity of questionnaire; Table S5: Discrimination validity of questionnaire.
