# Supplementary material for: Acceptance of Rubella-Containing Vaccine and Factors Associated with Acceptance among Women of Reproductive Age in China: A Cross-Sectional Study
Source: Vaccines (Basel). 2024 Oct 8;12(10):1153. doi: 10.3390/vaccines12101153 (PMC11511206; doi:10.3390/vaccines12101153)
Supplement: Supplementary file 1 [file vaccines-12-01153-s001.zip › vaccines-3201376-supplementary.pdf]

Supplemental Material

Table S1. Rubella knowledge score of respondents.

Characteristic	Total	Knowledge score [M (P_{25}-P_{75})]	Z/H	*P*-Value
Region			184.16	<0.001
Eastern region	365	5 (2-7)		
Central region	352	3 (0-5)		
Western region	366	7 (4-8)		
Northeast region	203	4 (2-6)		
Household registration			-3.00	0.003
Urban area	675	5 (2-7)		
Rural area	611	4 (1-7)		
Age			14.44	<0.001
15-24 year	264	4 (2-6)		
25-34 year	464	5 (2-7)		
35-49 year	558	4 (1-7)		
Occupation			13.46	<0.001
Medical practitioner	353	7 (5-8)		
Non-medical practitioner	933	4 (1-6)		
Education level			-10.83	<0.001
High school and below	533	3 (0-6)		
Bachelor’s degree and above	753	6 (3-7)		
Monthly income in CNY			51.18	<0.001
≤3000	205	3 (0-6)		

3001-5000	456	5 (2-7)		
5001-10000	384	5 (2-7)		
>10000	241	5 (3-7)		
Marital status			2.44	0.015
Married	900	4 (1-7)		
Unmarried	386	5 (3-7)		
Childbearing status			2.69	0.007
Yes	857	4 (1-7)		
No	429	5 (3-7)		
Number of children			-4.15	<0.001
One	473	5 (2-7)		
Two and above	384	4 (1-6)		
Adverse pregnancy history			-0.47	0.639
Yes	123	4 (1-7)		
No	734	4 (2-7)		
Rubella infection			3.90	<0.001
Yes	38	7 (4-8)		
No or not sure	1248	5 (2-7)		
Rubella vaccine			3.32	<0.001
Vaccinated	166	5 (3-7)		
Unvaccinated or not sure	1120	5 (2-7)		

Table S2. Comparison of RCV acceptance in the dimension of Thinking and Feeling, Social Processes, and Practical Issues.

Latent variables	Observed variables	Responses	Total		Willing to be vaccinated		χ^2	*P*-Value
			N	%	N	%		
Thinking and Feeling	Confidence in vaccine benefits	agree	834	64.9	709	85.0	99.93	<0.001
		disagree	452	35.1	272	60.2		
	Confidence in vaccine safety	agree	894	69.5	752	84.1	99.47	<0.001
		disagree	392	30.5	229	58.4		
	Confidence in vaccine importance	agree	957	74.4	801	83.7	113.71	<0.001
		disagree	329	25.6	180	54.7		
	Confidence in health workers	agree	1093	85.0	891	81.5	110.35	<0.001
		disagree	193	15.0	90	46.6		
Social Processes	Peer norms	agree	820	63.8	699	85.2	100.43	<0.001
		disagree	466	36.2	282	60.5		
	Family norms	agree	884	68.7	746	84.4	102.71	<0.001
		disagree	402	31.3	235	58.5		
	Health workers recommendation	agree	1015	79.0	839	82.7	108.27	<0.001
		disagree	271	21.0	142	52.4		
	National policy	agree	572	44.5	519	90.7	118.92	<0.001
		disagree	714	55.5	462	64.7		
Practical Issues	Vaccine accessibility	agree	1002	77.9	804	80.2	39.26	<0.001
		disagree	284	22.1	177	62.3		
	Convenience of vaccination	agree	1131	87.9	902	79.8	62.43	<0.001
		disagree	155	12.1	79	51.0		
	Service satisfaction	agree	1183	92.0	937	79.2	69.72	<0.001

	disagree	103	8.1	44	42.7		
Self-paid vaccination	agree	918	71.4	775	84.4	117.48	<0.001
	disagree	368	28.6	206	56.0		
Free vaccination	agree	1148	89.3	954	83.1	274.87	<0.001
	disagree	138	10.7	27	19.6		
Accept recommendation of health workers	agree	1125	87.5	939	83.5	256.31	<0.001
	disagree	161	12.5	42	26.1		

Table S3. Associations and values between latent variables and corresponding observed variables.

Latent variables	Observed variables	Values
Thinking and Feeling	Confidence in vaccine benefits	1 = totally disagree, 2 = disagree, 3=not sure, 4 = agree, 5 = totally agree
	Confidence in vaccine safety	
	Confidence in vaccine importance	
	Confidence in health workers	
	Knowledge	1 = low, 2 = middle, 3=high
Social Processes	Peer norms	1 = totally disagree, 2 = disagree, 3=not sure, 4 = agree, 5 = totally agree
	Family norms	
	Health workers recommendation	
	National policy	
Motivation	Vaccination willingness	1 = Yes, 0 = No
	Willingness to Recommend	
Practical Issues	Vaccine accessibility	

	Convenience of vaccination<br>Service satisfaction<br>Self-paid vaccination<br>Free vaccination<br>Accept recommendation of health workers	1 = totally disagree, 2 = disagree, 3=not sure, 4 = agree, 5 = totally agree
Covariate	Region	1 = Eastern region, 2 = Central region, 3 = Western region, 4 = Northeast region
	Occupation	1 = Medical practitioner, 2 = Non-medical practitioner
	Vaccination history	1 = Yes, 2 = No or not sure

Table S4. Factor loading and convergent validity of questionnaire.

Latent variables	Observed variables	Factor loading		CR	AVE
		Initial	Adjusted		
Thinking and Feeling	Confidence in vaccine benefits	0.745	0.748	0.856	0.551
	Confidence in vaccine safety	0.797	0.804		
	Confidence in vaccine importance	0.866	0.874		
	Confidence in health workers	0.736	0.731		
	Knowledge	0.554	0.501		
Social Processes	Peer norms	0.718	0.736	0.733	0.479
	Family norms	0.702	0.708		
	Health workers recommendation	0.640	0.627		
	National policy	0.191	\		
Motivation	Vaccination willingness	0.799	0.789	0.804	0.673

	Willingness to Recommend	0.836	0.850		
Practical Issues	Vaccine accessibility	0.487	0.430	0.817	0.443
	Convenience of vaccination	0.565	0.511		
	Service satisfaction	0.648	0.596		
	Self-paid vaccination	0.598	0.600		
	Free vaccination	0.813	0.837		
	Accept recommendation of health workers	0.870	0.892		

Note: AVE, average variance extraction; CR, critical ratio.

Table S5. Discrimination validity of questionnaire.

	Thinking and Feeling	Social Processes	Motivation	Practical Issues
Thinking and Feeling	0.514			
Social Processes	0.152	0.153		
Motivation	0.403	0.149	0.506	
Practical Issues	0.113	0.082	0.123	0.112
